# Assessing Temperature-Dependent DNA Cleavage by CRISPR-Cas9

**DOI:** 10.21769/BioProtoc.5463

**Published:** 2025-10-05

**Authors:** Alexa L. Knight, Jinping Luo, George P. Lisi

**Affiliations:** 1 Department of Molecular Biology, Cell Biology & Biochemistry, Brown University, Providence, RI, USA; 2 Transgenic & Genome Editing Facility, Brown University, Providence, RI, USA; 3 Brown University RNA Center, Providence, RI, USA

**Keywords:** CRISPR-Cas9, Thermophile, DNA cleavage, Enzyme, Endonuclease

## Abstract

The RNA-guided CRISPR-Cas9 endonuclease has been a transformative tool for laboratory biochemistry with huge potential as a precision therapeutic. This tool site-specifically cleaves double-stranded DNA following the recognition of a unique protospacer-adjacent motif (PAM). Activation of the protein–nucleic acid Cas complex has also been widely recognized to feature an allosteric mechanism dependent on structural remodeling and interdomain crosstalk. Biophysical methods have probed the impact of allosteric perturbations on cleavage and specificity of Cas9, with the aim of engineering enhanced Cas effectors. These studies include Cas9 from thermophilic organisms that edit at higher temperatures and are active in human plasma. Validation of biophysical insights has necessitated the quantitation of DNA cleavage in vitro and, subsequently, the adaptation of established protocols to encompass temperature-dependent function that is evident in extremophilic Cas systems, such as Cas9 from *Geobacillus stearothermophilus* and the mesophilic *Sp*Cas9. This protocol is advantageous for probing functional temperature ranges of DNA cleavage that can theoretically be applied to any Cas-RNP system.

Key features

• Builds upon the original Cas9 cleavage assays reported by Jinek et al. [1] to include the active temperature range of thermophiles.

• Validated for assessing the cleavage activity of both mesophilic and thermophilic Cas9 systems.

• Allows for qualitative and quantitative assessment of DNA cleavage across multiple physiological regimes.

• Can be adapted to assess cleavage at multiple genomic loci or with different PAM requirements.

• Assay can be completed in a single day.

## Graphical overview


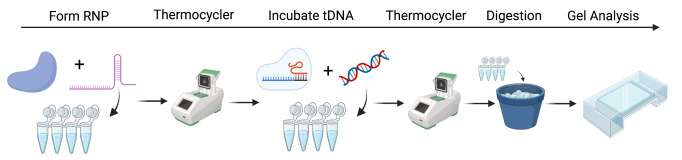


**CRISPR ribonucleoprotein (RNP) formation, cleavage assay preparation, and analysis. Step 1:** Incubate CRISPR-associated (Cas) protein with guide RNA (sgRNA) to form RNP. **Step 2:** Thermocycle to incorporate sgRNA. **Step 3:** At the desired temperature, incubate the target DNA (tDNA) that is complementary to the sgRNA to ensure cleavage. **Step 4:** Proteinase K digestion. **Step 5:** Gel analysis of DNA products.

## Background

The tunability of CRISPR-mediated DNA editing has revolutionized scientists' ability to probe the genetic origins of disease, to design novel therapeutics, and to dissect the molecular mechanisms of numerous genes. After more than a decade, advanced studies of CRISPR-associated (Cas) systems are still opening new avenues for precision medicines, biotechnology, agriculture, and basic biology [2,3]. Within the CRISPR-Cas toolkit, diverse endonucleases cleave double-stranded DNA via structural and dynamic fluctuations that impact the efficiency and specificity (i.e., off-target effects) of the system [4]. While protein engineering has produced high-specificity Cas variants with little-to-no off-target effects, a relatively understudied aspect of CRISPR biology remains the unique function of extremophiles capable of cleaving DNA at very high temperatures [5,6]. Such systems are beneficial for applications requiring prolonged Cas enzyme stability, an expanded scope of physiological activity, alternate PAM recognition motifs, or the potential to package Cas ribonucleoproteins (RNP) for therapeutic delivery [1,7]. As mechanistic studies of thermophilic CRISPR-Cas systems continue, along with efforts to enhance their already unique properties, fast and reliable methods for evaluating DNA cleavage over a very broad temperature range are essential. This protocol details a straightforward method for assessing CRISPR-Cas9 DNA cleavage at standard and elevated temperatures, providing a framework for qualitative and quantitative function evaluation.

## Materials and reagents

**Biological materials**

1. Mouse strain C57BL/6J (The Jackson Laboratory, strain #000664)

2. *Geo*Cas9 pET plasmid (Addgene, catalog number: 87700)

3. BL21(DE3) Competent *E. coli* (New England Biolabs, catalog number: C2527H)

**Reagents**

1. DreamTaq PCR Master Mixes (2×) (Thermo Fisher Scientific, catalog number: K1081)

2. Xpert directXtract PCR kit (BocaScientific, catalog number: GE60.0080)

3. UltraPure^TM^ DNase/RNase-free distilled water (Thermo Fisher Scientific, catalog number: 10977015)

4. UltraPure^TM^ 1 M Tris-HCl buffer, pH 7.5 (Thermo Fisher Scientific, catalog number: 15567027)

5. MgCl_2_ (1 M) (Thermo Fisher Scientific, catalog number: AM9530G)

6. Potassium chloride (KCl), 1 M aq. soln., RNAse-free (Thermo Fisher Scientific, catalog number: J62422.AE)

7. Dithiothreitol (DTT) (Thermo Fisher Scientific, catalog number: R0861)

8. Glycerol (molecular biology) (Fisher BioReagents^TM^, Fisher Scientific, catalog number: BP229-1)

9. 25 nmol DNA oligo (Tnnt2-F) 5′- CAAAGAGCTCCTCGTCCAGT -3′ (Genewiz)

10. 25 nmol DNA oligo (Tnnt2-R) 5′- ATGGACTCCAGGACCCAAGA -3′ (Genewiz)

11. Tnnt2_21bp gRNA or Tnnt2_23bp gRNA (sequences described in Section C) (GenScript)

11. Tris-acetate-EDTA (TAE) buffer (50×) (Thermo Fisher Scientific, catalog number: B49)

12. Agarose LE (molecular biology grade) (GoldBio, catalog number: A-201-100)

13. Proteinase K solution 5 mL (20 mg/mL) (Viagen, catalog number: 501-PK)

14. GelRed^®^ nucleic acid gel stain (10,000× in water) (Biotium, catalog number: 41003)

15. Custom mRNA synthesis (5 nmol) (GenScript)

16. Tris-HCl (Thermo Fisher Scientific, catalog number: 15506017)

17. HEPES (Thermo Fisher Scientific, catalog number: A14777.09)

18. Imidazole (Fisher Scientific, catalog number: AAA1022122)

19. TCEP-HCl (Fisher Scientific, catalog number: PI20491)

20. NaCl (Fisher Scientific, catalog number: BP358-1)

21. Terrific broth (Fisher Scientific, catalog number: 22-711-022)

22. PMSF protease inhibitor (Thermo Fisher Scientific, catalog number: 36978)

**Solutions**

1. 5× in vitro cleavage reaction buffer (see Recipes)

2. *Geo*Cas9 Ni-affinity lysis buffer for recombinant protein purification (see Recipes)

3. *Geo*Cas9 Ni-affinity elution buffer for recombinant protein purification (see Recipes)

4. *Geo*Cas9 heparin affinity “buffer A” for recombinant protein purification (see Recipes)

5. *Geo*Cas9 heparin affinity “buffer B” for recombinant protein purification (see Recipes)

6. *Geo*Cas9 TEV cleavage buffer (see Recipes)

7. *Geo*Cas9 size-exclusion chromatography buffer (see Recipes)

8. *Geo*Cas9 protein storage (see Recipes)

**Recipes**

**1. 5× in vitro DNA cleavage reaction buffer**

**Table:** 

Reagent	Final concentration	Quantity or volume
Glycerol ^a^	25% (w/v)	2.5 g
1 M Tris-HCl, pH 7.5	100 mM	1.0 mL
1 M KCl	500 mM	5.0 mL
1 M MgCl_2_	25 mM	0.25 mL
0.1 M DTT ^b^	5 mM	0.5 mL
Ultrapure distilled water		Up to 10 mL

Store the reaction buffer without DTT at 4 °C and use it within 3 months.

^a^ Glycerol is weighed in a 15 mL conical tube, then the other components are added to the tube.

^b^ DTT is unstable in solution long term. Only freshly prepared DTT solutions should be used. To prepare a 0.1 M DTT solution, dissolve 15.5 mg of DTT (MW 154.25 g/mol) powder in 1 mL of ultrapure distilled water.

**2. *Geo*Cas9 Ni-affinity lysis buffer for recombinant protein purification**

**Table:** 

Reagent	Final concentration	Quantity or volume
H_2_O	n/a	1,000 mL
Tris-HCl, pH 7.5	50 mM	7.88 g
NaCl	500 mM	29.22 g
TCEP	0.5 mM	0.143 g
Imidazole	10 mM	0.681 g
Total		1,000 mL

**3. *Geo*Cas9 Ni-affinity elution buffer for recombinant protein purification**

**Table:** 

Reagent	Final concentration	Quantity or volume
H_2_O	n/a	1,000 mL
Tris-HCl, pH 7.5	50 mM	7.88 g
NaCl	500 mM	29.22 g
TCEP	0.5 mM	0.143 g
Imidazole	250 mM	17.02 g
Total		1,000 mL

**4. *Geo*Cas9 heparin affinity “buffer A” for recombinant protein purification**

**Table:** 

Reagent	Final concentration	Quantity or volume
H_2_O	n/a	1,000 mL
Tris-HCl, pH 7.5	20 mM	3.15 g
NaCl	150 mM	8.77 g
TCEP	1 mM	0.287 g
Total		1,000 mL

**5. *Geo*Cas9 heparin affinity “buffer B” for recombinant protein purification**

**Table:** 

Reagent	Final concentration	Quantity or volume
H_2_O	n/a	1,000 mL
Tris-HCl, pH 7.5	20 mM	3.15 g
NaCl	1.3 M	75.97 g
TCEP	1 mM	0.287 g
Total		1,000 mL

**6. *Geo*Cas9 TEV cleavage buffer**

**Table:** 

Reagent	Final concentration	Quantity or volume
H_2_O	n/a	950 mL
Tris-HCl, pH 7.5	20 mM	3.15 g
NaCl	250 mM	14.61 g
TCEP	1 mM	0.287 g
Glycerol	5% (v/v)	50 mL
Total		1,000 mL

**7. *Geo*Cas9 size-exclusion chromatography buffer**

**Table:** 

Reagent	Final concentration	Quantity or volume
H_2_O	n/a	950 mL
Tris-HCl, pH 7.5	20 mM	3.15 g
NaCl	250 mM	14.61 g
TCEP	1 mM	0.287 g
Glycerol	5% (v/v)	50 mL
Total		1,000 mL

**8. *Geo*Cas9 protein storage**

**Table:** 

Reagent	Final concentration	Quantity or volume
H_2_O	n/a	950 mL
HEPES, pH 7.5	20 mM	4.76 g
NaCl	150 mM	8.77 g
TCEP	1 mM	0.287 g
Glycerol	5% (v/v)	50 mL
Total		1,000 mL

*Note: In Recipes 2–8, buffer components are weighed and added to 1,000 mL or 950 mL of H_2_O, and pH readings are taken. The pH is adjusted accordingly with concentrated stocks of HCl or NaOH.*

**Laboratory supplies**

1. NucleoSpin Gel and PCR Clean-up, Mini kit for gel extraction or PCR clean up (Macherey-Nagel, catalog number: 740609.50)

2. TempAssure 0.2 mL PCR 8-tube strip, Opti. Hinge-strip cap (USA Scientific, catalog number: 1402-1800)

3. Thin-walled, frosted lid, RNase-free PCR tubes (0.2 mL) (Invitrogen, catalog number: AM12225)

4. Amicon^®^ Ultra centrifugal filter, 10 kDa MWCO (Sigma-Aldrich, catalog number: UFC901008)

5. SnakeSkin^TM^ dialysis tubing 10 kDa (Thermofisher, catalog number: 88245)

## Equipment

1. C1000 Touch Thermal Cycler with Dual 48/48 Fast Reaction Module (Bio-Rad, catalog number: 1851148)

2. Nanodrop Lite Spectrophotometer with Printer (Thermo Fisher Scientific, catalog number: 840281500)

3. Owl^TM^ A2 Large Gel Systems (Thermo Fisher Scientific, catalog number: A2-BP)

4. Owl^TM^ EC-105 Compact Power Supply (Thermo Fisher Scientific, catalog number: 105ECA-115)

5. GelDoc-It^®^ 310 Manual Lens W/LM-26 Transilluminator and UVP Framework software (UVP Inc)

6. NGC Quest 10 Plus Chromatography System (Bio-Rad Laboratories, catalog number: 7880003)

7. EconoFit Profinity IMAC Columns, Ni-charged, 5 × 5 mL (Bio-Rad Laboratories, catalog number: 12009300)

8. HiLoad 26/600 Superdex 200 pg (Cytiva, catalog number: 28989336)

9. Fisherbrand^TM^ Model 120 Sonic Dismembrator (Fisherbrand, catalog number: FB120110)

10. Allegra X-30R Clinical 220–240 V, 50/60 Hz Centrifuge, IVD (Beckman Coulter, catalog number: B06321)

11. Avanti J-E Series Floor Centrifuge

12. Affi-Gel Heparin Gel (Bio-Rad Laboratories, catalog number: 1536173)

## Software and datasets

1. ImageJ (Version 1.53). ImageJ is a public-domain Java image processing program inspired by NIHImage for Macintosh. It runs either as an online applet or as a downloadable application on any computer with a Java 1.4 or later virtual machine. Free downloads for Windows, Mac OS, Mac OS X, and Linux are available at https://imagej.net/ij/download.html

2. GraphPad Prism (Version 9)

*Note: Any graphing software can be used to generate the outputs.*

## Procedure

**A. Prepare DNA substrates**

The DNA substrate contains a single Cas9 targeting site. The DNA substrate can be synthetic DNA fragments, plasmid DNA, or column-purified PCR products amplified from plasmid DNA or genomic DNA, depending on the lab resources. Here, we choose to make the DNA substrate with PCR from mouse genomic DNA, where the targets of interest are broadly available.

1. Extract genomic DNA from ear punch tissue of C57BL/6J mice using the Xpert directXtract PCR Kit following full instructions. In short, add 50 μL of lysis solution consisting of 35 μL of ultrapure distilled water, 10 μL of Xpert directXtract buffer A, and 5 μL of Xpert directXtract buffer B to cover a 1–2 mm mouse tissue in a 0.2 mL PCR tube. Incubate at 37 °C for 10 min to lyse the tissue and then incubate at 95 °C for 10 min to terminate the reaction. Finally, cool the tube to room temperature. Centrifuge the tube at 14,000–16,000× *g* for 1 min to pellet tissue debris and transfer 35 μL of the cleared supernatant to a new 0.2 mL PCR tube to be used as the PCR template that is suitable to amplify DNA up to 5 kb. The genomic DNA can be stored at -20 °C for at least 3 months.

2. PCR amplification of the target region. The optimal length of the DNA substrate is 0.5–1.0 kb, and the optimal cleavage site is located near the center of the amplified DNA sequence for the in vitro cleavage assay. Design specific PCR primers with the online tool NCBI Primer-BLAST (https://www.ncbi.nlm.nih.gov/tools/primer-blast/index.cgi).

Set up a 20 μL PCR reaction (32 repeats) with DreamTaq PCR Master Mix (2×) according to the product instructions.

Ultrapure water    8.0 μL

DreamTaq PCR Master Mixes (2×) 10.0 μL

Forward primer (Tnnt2-F, 10 μM)  0.5 μL

Reverse primer (Tnnt2-R, 10 μM)  0.5 μL

Mouse genomic DNA lysate  1.0 μL

*Note: Prepare a master mix of 32 PCR reactions, each 20 μL in volume, and then distribute it into 8-tube PCR strips.*

Run PCR with the following PCR program:

a) 95 °C, 2 min

b) 35 cycles of:

95 °C, 30 s

60 °C, 30 s

72 °C, 60 s

c) 72 °C, 10 min

d) 4 °C, hold

*Note: Adjust the PCR annealing temperature between 55 and 62 °C to generate a specific band. The PCR extension time of 1 min is suitable for amplicons under 2 kb.*

Combine the PCR products (640 μL) from the 24 reactions and use 3 μL to run a 1% agarose gel to verify the amplified DNA for a single band. Take gel images for records and analyses.

3. Purify PCR products and measure DNA concentration. Purify the PCR products using the NucleoSpin Gel and PCR clean-up kit following the instructions. In short, add 1,280 μL of the NTI binding buffer to the 640 μL of the PCR products, mix well, and then evenly load the mixture into two columns to start purification. After washing three times with 700 μL of NT3 buffer, elute the DNA with 50 μL of elution buffer (5 mM Tris-HCl, pH 8.5) per column to harvest a total of 100 μL of DNA. Take 1 μL of the eluted DNA and measure the concentration using the Nanodrop Lite Spectrophotometer. The DNA concentration is in the range of 120–160 ng/μL. Store DNA substrate in a -20 °C freezer.

*Note: Purified double-stranded DNA of good quality has an A_260_/A_280_ of 1.85–1.90.*

**B. Express and purify *Geo*Cas9 protein in-house**

The *Geo*Cas9 plasmid was acquired from Addgene (#87700) and transformed into BL21 (DE3) cells. The protein was expressed in 1 L of TB media as previously described [5,7]. The purification of *Geo*Cas9 is described in detail below.

*Note: Keep all solutions and reagents on ice and at 4 °C.*

1. Resuspension and lysing of cells. Resuspend the centrifuged cell pellet in 30 mL of lysis buffer with 1 mL of 100 mM PMSF. Lyse cells by sonication (4× pulse: 5 s on, 5 s off, time: 2 m 30 s per cycle, amplitude: 60%) on ice. Remove cell debris by centrifugation at 15,500 rpm (29,070× *g*) for 30 min. Collect supernatant in a Falcon tube (also take a 15 μL sample of supernatant and pellet for SDS-PAGE gel analysis).

2. Purification of His-tagged *Geo*Cas9 by Ni cartridge. Place FPLC line A into lysis buffer (Recipe 2) and line B into elution buffer (Recipe 3), and flush lines. Zero the UV baseline to the lysis buffer. Equilibrate the Ni-NTA cartridge with 2× the column volume of lysis buffer. Equilibration can be done by hand with a syringe or while attached to FPLC. If equilibrated while attached to the FPLC, remove the cartridge from the FPLC and use a syringe with a 0.45 μm filter to flow the supernatant onto the Ni-NTA column. You may also flow the supernatant over the cartridge using the FPLC pump lines, but we have found that flowing by hand is more effective. Attach the Ni-NTA column to the FPLC and wash the column with 70 mL of lysis buffer. Elute *Geo*Cas9 with a 0%–100% gradient of elution buffer for 150 mL and collect the protein fractions (5 mL each). Confirm the presence of protein by SDS-PAGE, take fractions with eluted *Geo*Cas9, and buffer exchange into heparin column buffer A. Buffer exchanging involves replacing the buffer solution with another to alter solution properties; in this case, exchanging a high salt solution for a low salt solution to prepare the sample for the heparin column. Buffer exchange can be accomplished using an Amicon spin concentrator, allowing for rapid concentration of the protein sample to a low volume, followed by repeated dilution and reconcentration with a new buffer, to ensure a uniform solution. Another method of buffer exchange is equilibrium dialysis, though this often requires overnight treatment of the sample (at 4 °C for Cas9) and proper dialysis tubing (see Laboratory supplies). The method of buffer exchange depends on the experimental planning constraints and protein stability; however, both are effective methods. After the protein is buffer exchanged, concentrate using a 10 kDa molecular weight cut off (MWCO) Amicon spin concentrator. The volume you choose to concentrate will depend on the loop volume of your FPLC. We use a 10 mL loop to inject onto the heparin column.

3. Purification of *Geo*Cas9 by the heparin cartridge. Collect ~5 mL of heparin buffer A with a syringe, add heparin buffer A (Recipe 4) to line 1 and heparin buffer B (Recipe 5) to line 2, and flush the FPLC system. With the column bypassed on the flowpath, inject ~3 mL of heparin buffer A with a syringe and run 20 mL of heparin buffer A through the injection loop. This equilibrates the injection loop into the buffer our protein is in. Open the injection loop and place the heparin column in the instrument flowpath. Run buffer A over the column at a rate of 5 mL/min. Turn on the UV detector and zero the instrument baseline to buffer A. Using a syringe, load the 10 mL of *Geo*Cas9 sample onto the column via the injection loop. Wash the column with ~70 mL of heparin buffer A. Elute *Geo*Cas9 with a 0%–100% gradient of heparin buffer B for a total of 150 mL and collect the protein fractions (1 mL each). Confirm the presence of protein by SDS-PAGE and dialyze FPLC fractions containing *Geo*Cas9 along with TEV protease (1 mL, 100 μM) against 2 L of TEV cleavage buffer (Recipe 6) overnight at 4 °C. The next morning, remove the protein from the dialysis tubing (save 15 μL for SDS-PAGE) and buffer exchange the solution into size-exclusion chromatography buffer (Recipe 7). Concentrate the protein to a volume of 10 mL using a 10 kDa MWCO Amicon spin concentrator.

4. Purification of *Geo*Cas9 by Superdex 200 size-exclusion column. Equilibrate the HiLoad 26/600 Superdex 200 column with 330 mL of *Geo*Cas9 size-exclusion chromatography buffer at a flow rate of 2.6 mL/min on an FPLC. Load the 10 mL protein sample onto the size-exclusion column via the injection loop and purify with a flow rate of 2.6 mL/min. Collect fractions (2 mL each). Confirm the presence and final purity of protein by SDS-PAGE. If additional gel bands, indicative of impurities, are visible, use a second column step (see step B2) to further purify. Combine the final protein fractions and buffer exchange into *Geo*Cas9 storage buffer (Recipe 8). Concentrate the protein into 1.5 mL aliquots using a 10 kDa MWCO Amicon spin concentrator. Flash freeze aliquots and store at -80 °C. Final concentration typically ranges from 50 to 100 μM in ~5 mL.

*Note: The additional Ni-NTA column step is not often needed; however, it can be useful if size-exclusion chromatography does not separate all leftover TEV protease. The buffer exchange that takes place at multiple stages throughout the protocol is important for the removal of unwanted salts, reagents, or components from prior steps and aids in maintaining protein stability, activity, and solubility.*

**C. *Geo*Cas9 single-guide RNA preparation**

*Geo*Cas9 single-guide RNA (sgRNA) targeting the mouse *Tnnt2* gene with the PAM (CCTCCAAA) and a 21nt (i) or 23nt (ii) spacer (the leading nucleotides in lowercase in the following sequences) was ordered as 5 nmol customized CRISPR RNA from GenScript. The lyophilized powder was received and resuspended in 50 or 100 μL of ultrapure distilled water for a stock at 100 or 50 μM, as suggested on the tube. The final concentration was further determined using the Nanodrop Lite Spectrophotometer. 5 μL per aliquot was stored at -80 °C.

i. gcacctaccttctggatgtacGTCATAGTTCCCCTGAGAAATCAGGGTTACTATGATAAGGGCTTTCTGCCTAAGGCAGACTGACCCGCGGCGTTGGGGATCGCCTGTCGCCCGCTTTTGGCGGGCATTCCCCATCCTT

ii. ttgcacctaccttctggatgtacGTCATAGTTCCCCTGAGAAATCAGGGTTACTATGATAAGGGCTTTCTGCCTAAGGCAGACTGACCCGCGGCGTTGGGGATCGCCTGTCGCCCGCTTTTGGCGGGCATTCCCCATCCTT

**D. Assay *Geo*Cas9 DNA cleavage in vitro**

1. Collect the following reagents for the cleavage reaction:

a. 14 μM *Geo*Cas9 (M.W. 126.95kDa), stored at -80 °C

b. 28.5 μM Tnnt2_21bp gRNA (MW, 44.52 kDa) or Tnnt2_23bp gRNA (MW, 45.13 kDa), stored frozen at -80 °C

c. 0.51 μM DNA substrate (151.8 ng/μL, 479 bp, 296.02 kDa, A_260/280_ = 1.85), stored frozen at -20 °C or -80 °C

2. Prepare the RNP complex of *Geo*Cas9 and guide RNA.

a. Set a 200 μL PCR tube on ice and add the stock *Geo*Cas9 recombinant protein and the stock guide RNA according to Table 1 to form a 3 μM RNP complex.

**Table 1.** Components for Cas9-RNP formation

Stock components	RNP concentration	Volume (μL)
14 μM *Geo*Cas9	3 μM	2.15
28.5 μM sgRNA	3 μM	1.05
1× reaction buffer	N/A	6.80
Total RNP^a^		10.0

^a^ Total RNP volume is based on the planned reaction numbers and the working RNP concentration.

b. Invert the PCR tube, briefly spin, and then incubate the tube on a thermocycler at 37 °C or 60 °C for 10 min.

c. Store on ice until use.

3. Prepare the in vitro cleavage reactions.

a. Calculate the total number of reactions (e.g., six reactions) using Table 2.

**Table 2.** Cas9 reaction mixture components

Reaction	RNP conc. (μM)	3 μM *Geo*Cas9 RNP (μL)	0.5 μM DNA substrates ^a^ (μL)	5× cleavage reaction buffer ^b^ (μL)	Nuclease-free H_2_O (μL)	Total volume (μL)
1	0.1	0.33	1	2	6.67	10
2	0.2	0.67	1	2	6.33	10
3	0.3	1	1	2	6	10
4	0.6	2	1	2	5	10
5	0.9	3	1	2	4	10
6	0	0	1	2	7	10

^a^ Different batches of DNA substrate at concentrations of 120–160 ng/μL were prepared, and ~150 ng was used per reaction.

^b^ The buffer is described in Recipe 1.

b. Set up one 8-strip PCR tube on ice and add the components in Table 2, following the order: water, 5× cleavage buffer, DNA substrates, and RNP.

*Note: To facilitate the process, prepare a master mixture with 6.5 μL of 0.5 μM DNA substrate and 13 μL of 5× cleavage reaction buffer. Then, add 3 μL of this mixture to each reaction, rather than individually adding DNA substrate and 5× cleavage reaction buffer.*

c. Invert the PCR tubes and transiently spin.

d. Warm the thermocycler to the cleavage reaction temperature (e.g., 37 °C) before placing the tubes inside.

*Notes:*

*1. When incubating a reaction using a thermocycler, set up the thermocycler lid temperature 5 °C above the reaction temperature to prevent the reaction mixture from drying due to evaporation.*

*2. For a comparison of cleavage reactions at different temperatures (e.g., 37 and 60 °C), duplicates (or triplicates for three different temperatures: 37, 60, and 75 °C) of a reaction can be prepared, and incubation will be performed at each cleavage reaction temperature.*

e. Put the reactions of 8-strip tubes on the prewarmed thermocycler and incubate for 30 min.

f. After cleavage incubation, place the tubes on ice, add 1 μL of proteinase K (20 mg/mL) to each reaction, and incubate at 56 °C for 10 min.

g. Place the tubes on ice and add 2 μL of 6× DNA loading buffer.

**Figure 1.**
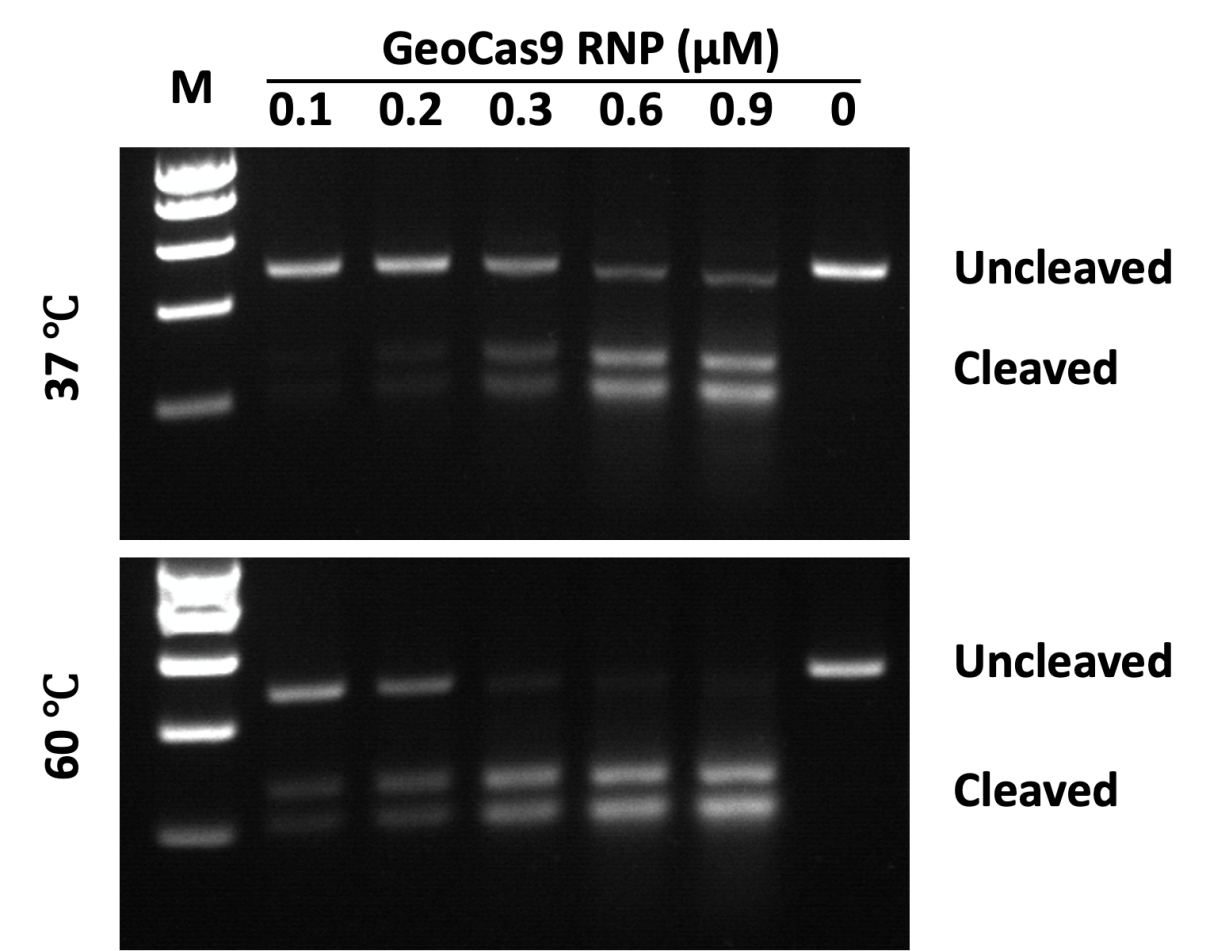
Visualization of temperature-dependent DNA cleavage. *Geo*Cas9 RNPs incubated at 37 °C (upper) or 60 °C (lower) for 10 min, after which the ribonucleoproteins (RNPs) were used in cleavage reactions carried out at 37 °C for 30 min. The appearance of lower molecular weight bands on the gel indicates successful cleavage. RNP concentrations range from 0.1 to 0.9 μM at each temperature tested, as indicated in Table 2. The rightmost lanes of each gel marked as (0) indicate negative controls with no RNP added. Molecular weight markers (M) are 1,000, 800, 600, 400, and 200 base pairs (top to bottom).

4. Gel analysis

a. Prepare a 2% agarose gel in 1× TAE buffer with the addition of GelRed^®^ DNA binding dye (10,000×).

b. Load 6 μL of each cleavage reaction mixed with DNA loading buffer to run the gel.

c. Run the gel at 300 mA for approximately 25 min.

d. Image the gel with the UVP gel imaging system and save as a .TIF file (Figure 1).

e. Analyze the bands with the ImageJ software (see Data analysis).

## Data analysis

**Figure 2.**
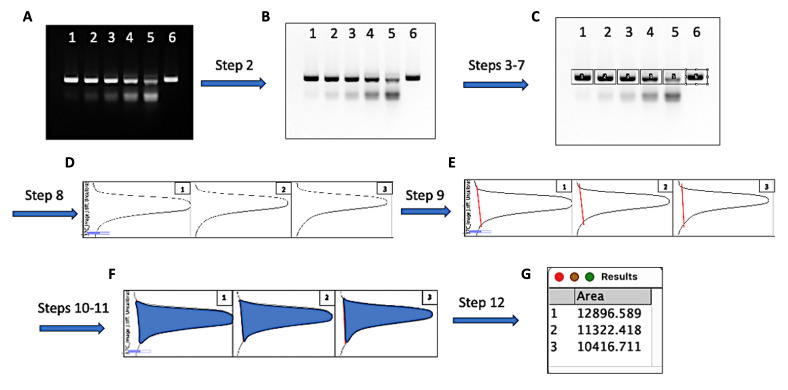
Measuring the density of uncleaved and cleaved DNA bands with ImageJ. Quantitative analysis of *Geo*Cas9 cleavage can be carried out according to the workflow described above. The steps in the figure correspond to those in the data analysis section. Representative snapshots of the analytical steps are provided in cartoon form. **(A)** The original gel image in which lanes 1–6 were loaded with 5 μL of the finished *Geo*Cas9 RNP/DNA cleavage reactions corresponding to 0.1–0.9 μM RNP and a negative control as described in Table 2. **(B)** Converted gel image by the ImageJ function *Invert LUT*. **(C)** Uncleaved DNA bands in each lane were enclosed by rectangles in the order of lanes **(D)** Plots were generated by applying *Plot Lanes* after the DNA bands were selected (only the first three lanes’ plots are shown as representatives). **(E)** The measuring area was closed by drawing a straight line (red) at the base of each plot peak with the *Straight* line tool. **(F)** The highlighted plot area after applying the *Wand (tracing)* tool by clicking inside the closed peak spot of each plot. **(G)** Automatically generated tab with the area measurements after applying *Wand (tracing)* tool in the closed peaks; the tab can be saved, and the measurements can be copied for later analysis.

1. Measure the uncleaved and cleaved DNA bands’ density using the following steps and ImageJ software (Figure 2). First, open the image file using *File > Open* in ImageJ.

2. Apply *Invert LUT* to convert the bright DNA bands into dark bands against a white gel background.

3. Choose the *Rectangular Selection* tool from the ImageJ toolbar. Draw a rectangle around the DNA band in the first lane. Center the rectangle over the DNA band from left to right.

4. After drawing the first rectangle, go to *Analyze > Gels > Select First Lane* to set the rectangle in place. The first rectangle will now be highlighted and display a “1” in its center.

5. Use your mouse to click and hold in the middle of the rectangle on the first lane and drag it over to the next lane.

*Note: ImageJ will automatically align the rectangle on the same vertical axis as the first rectangle in the next step.*

**Figure 3.**
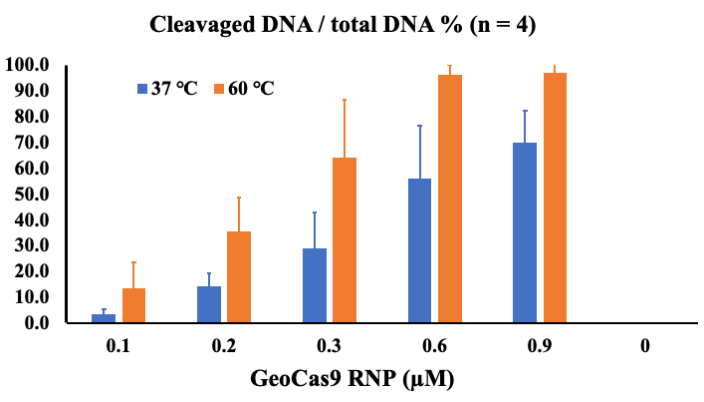
Calculation of the percentage of cleaved DNA from total DNA substrate in cleavage reactions using different concentrations of *Geo*Cas9-RNP. Half of each reaction volume (6 μL, including 1 μL of 6× DNA loading buffer) was loaded onto the gel. The areas of uncleaved DNA bands and cleaved double bands in each gel lane were measured using ImageJ as described. To present the cleavage efficiency, the percentage of cleaved DNA from total DNA was calculated by dividing the area of the cleaved bands by the total area of the uncleaved and cleaved bands. The bar graph represents the average value of four independent experiments, with errors represented as standard deviation.

6. Go to *Analyze > Gels > Select Next Lane* to set the rectangle in place over the second lane. A “2” will appear in the second rectangle placed.

7. Repeat steps 5 and 6 for each subsequent lane on the gel to set the rectangles in place.

8. Finish the rectangle in place on the last lane and select *Analyze > Gels > Plot Lanes* to draw a profile plot of each DNA band covered by the rectangles.

9. Choose the *Straight-Line* tool from the ImageJ toolbar. Draw a line across the base of the peak to enclose the peak in each profile plot.

10. When each peak has been closed off at the base with the *Straight-Line* tool, select the *Wand* tool from the ImageJ toolbar.

11. Apply the *Wand* tool by clicking the area inside the enclosed peak to get the results in a newly shown window.

12. Apply *File > Save* to save the results in an Excel file format.

13. Calculate the percentage of cleaved DNA over the total DNA load, as outlined in Figure 3.

**Discussion**

In these assays, we tested different concentrations of the *Geo*Cas9-RNP (0.1–0.9 μM) with a fixed concentration of double-strand DNA substrate (50 nM) in a 10 μL cleavage reaction. The *Geo*Cas9-RNP was formed by incubation of 3 μM *Geo*Cas9 and 3 μM sgRNA at 37 *°*C for 10 min before initiating the cleavage reaction, which was performed at different temperatures for 30 min. To terminate the reaction, 1 μL of 20 mg/mL proteinase K was added with subsequent incubation at 56 *°*C for 10 min. After adding 2 μL of 6× DNA loading buffer to each reaction, a 6 μL mixture of the reaction/proteinase K/DNA loading buffer was loaded onto the agarose gel to separate the uncleaved and cleaved DNA fragments. Gel images were taken for cleavage analysis using ImageJ.

The current protocol can be used to directly test whether a new source of Cas9 or guide RNA is working. There are challenges for comparison and quantitation, where the amount of DNA and the proportion of DNA to Cas9-RNP in the reaction may be crucial to achieving the experimental goals. In our tests, the size of the DNA substrate is 479 bp with 53% GC content, where double-strand cleavage resulted in 269 and 210 bp bands. Under the current reaction conditions, 600 nM or higher concentration of *Geo*Cas9-RNP to 50 nM DNA substrate in a 10 μL reaction resulted in close to 100% cleavage by incubation at the optimal temperature of *Geo*Cas9 (60 °C). We expect that the extension of the incubation time or the reduction of the DNA substrate would achieve 100% cleavage.

Quantitation with ImageJ requires an optimal quality of the DNA image. The DNA bands should be well separated, with condensed, sharp signals on the gel, and should not be overexposed during imaging. These standards will generate better accuracy in the measurement of the DNA quantity. The expanded DNA bands mainly cause a significant deviation of the total calculated area. This can be reduced by loading a lower amount of the DNA (e.g., 1/3 of the reaction volume, or ~50 ng DNA/lane) and improving the gel resolution of the DNA fragment separation with a higher percentage of the gel based on the shortest DNA fragment and running for a longer time. The minimum size of the ImageJ rectangles should completely enclose the bands to be measured, with care to ensure no overlap between the lanes.

## Validation of protocol

This protocol is routinely applied to quality-control new batches of recombinant Cas9 proteins prepared in-house. This protocol (or parts of it) has also been used and validated in Belato et al. [5]. Structural and Dynamic Impacts of Single-atom Disruptions to Guide RNA Interactions within the Recognition Lobe of *Geobacillus stearothermophilus* Cas9. *eLife*. 2025. DOI: 10.7554/eLife.99275.1 (see Figure S17 and Figure S18).

Previous work from which the protocol was developed and modified includes Belato et al. [6]. Disruption of Electrostatic Contacts in the HNH Nuclease from a Thermophilic Cas9 Rewires Allosteric Motions and Enhances High-temperature DNA Cleavage. *J. Chem. Phys*. 2022. 157. 225103-225113 (see Figure 4) and Harrington et al. [7]. A Thermostable Cas9 with Increased Lifetime in Human Plasma. *Nature Commun.* 2017. 8, DOI: 10.1038/s41467-017-01408-4 (see Figures 4 and 5).
